# Diffuse large B‐cell lymphoma presenting with masses in the pineal and adrenal glands

**DOI:** 10.1002/ccr3.2016

**Published:** 2019-01-29

**Authors:** Cem Bilgin, Baris Korkmaz, Esra Soylu, Hulya Ozturk, Kerem Ozturk

**Affiliations:** ^1^ Department of Radiology Uludag University Faculty of Medicine Bursa Turkey; ^2^ Department of Pathology and Laboratory Medicine Uludag University Faculty of Medicine Bursa Turkey

**Keywords:** adrenal gland, diffuse large B‐cell lymphoma, hydrogen MR spectroscopy, magnetic resonance imaging, pineal gland

## Abstract

Magnetic resonance imaging (MRI) may offer several potential advantages in the evaluation of lymphoma with the additive value of H^1^‐MRS for differential diagnosis. Even though lymphoma has unique imaging findings on CT and multiparametric MRI, definite diagnosis must be thoroughly established by histopathological examination.

A 39‐year‐old man presented to our emergency department due to worsening headaches for 2 weeks. On admission, there were no palpable superficial lymph nodes, nor palpable liver and spleen. Laboratory tests were within normal levels except that LDH was 350 mU/mL. MRI scan showed a pineal tumor, which was uniformly isointense on the T2‐weighted image (Figure 1), and showed homogeneous enhancement (Figure 2). The axial T2‐weighted MRI demonstrated frankly abnormal bilateral volumes of adrenal masses (Figure 3). Single‐voxel hydrogen MR (H^1^‐MR) spectroscopy showed an increase in choline/creatine ratio in the adrenal lesion (Figure 4). A CT‐guided biopsy of the right adrenal gland lesion was performed. Hematoxylin‐eosin stain showed diffuse infiltration of neoplastic lymphoid cells (Figure 5). Bone marrow biopsy confirmed a diagnosis of diffuse large B‐cell lymphoma (DLBCL) (Figure 6). Staging CT and ultrasound examinations demonstrated no manifestations outside these locations. The patient died 8 months after diagnosis due to progressive disease.

**Figure 1:**
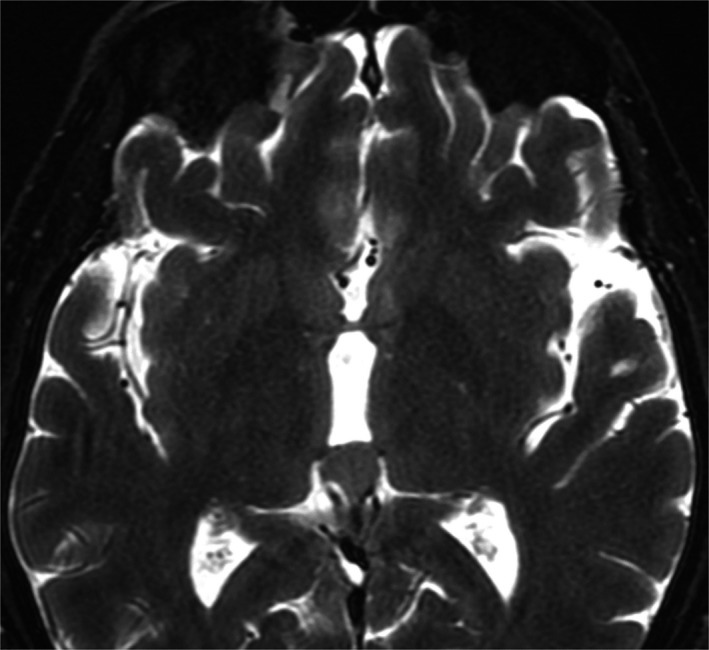
Diagnosis of diffuse large B‐cell lymphoma in a patient with masses in the pineal and adrenal glands

**Figure 2:**
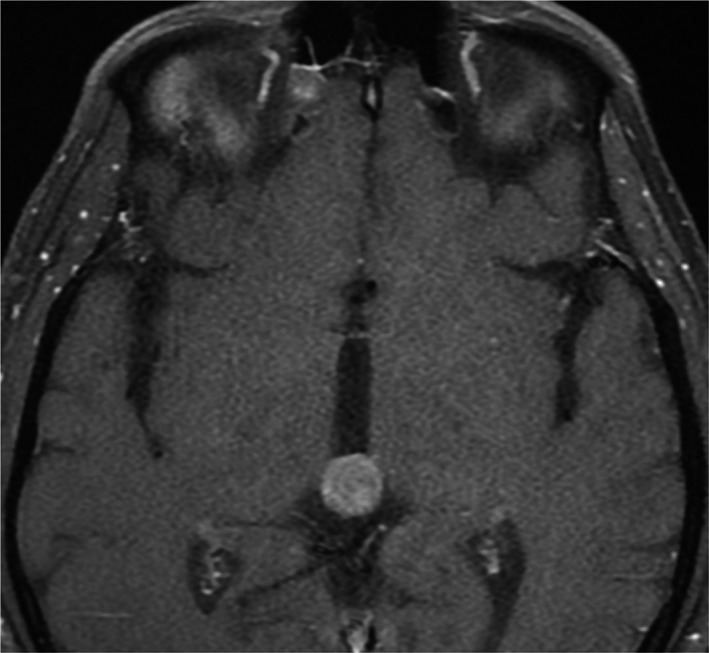
Diagnosis of diffuse large B‐cell lymphoma in a patient with masses in the pineal and adrenal glands

**Figure 3:**
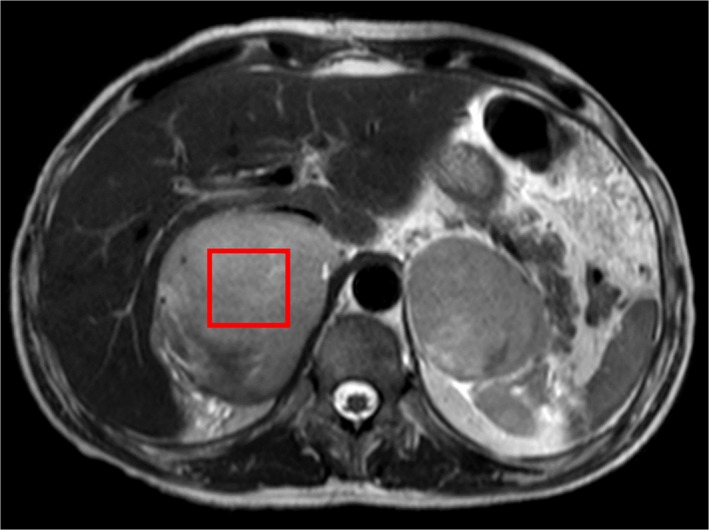
Diagnosis of diffuse large B‐cell lymphoma in a patient with masses in the pineal and adrenal glands

**Figure 4:**
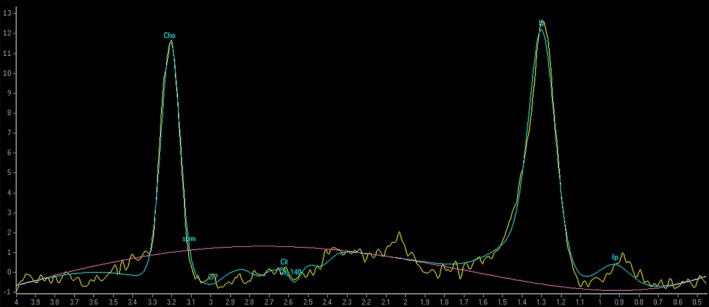
Diagnosis of diffuse large B‐cell lymphoma in a patient with masses in the pineal and adrenal glands

**Figure 5:**
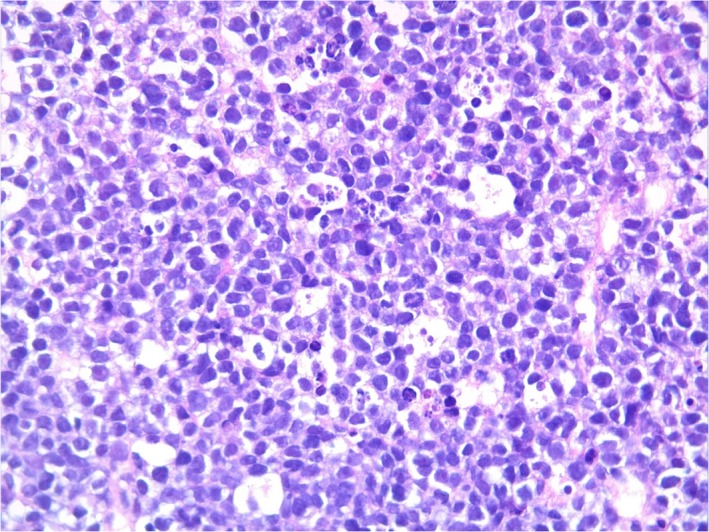
Diagnosis of diffuse large B‐cell lymphoma in a patient with masses in the pineal and adrenal glands

**Figure 6:**
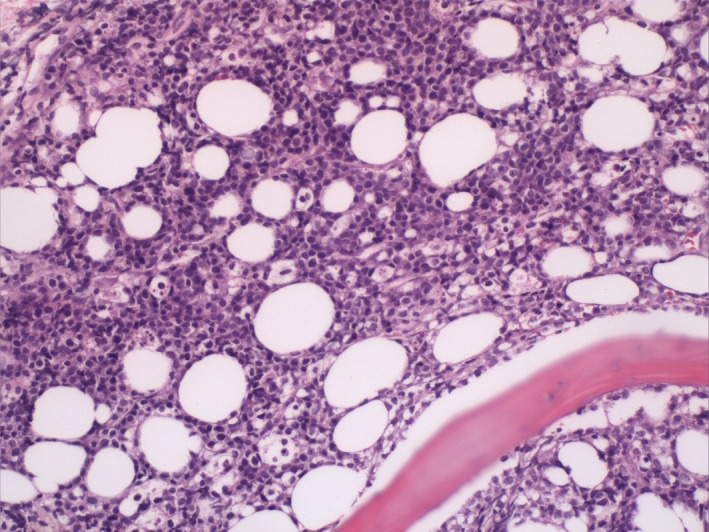
Diagnosis of diffuse large B‐cell lymphoma in a patient with masses in the pineal and adrenal glands

Diffuse large B‐cell lymphoma spreads to the pineal and adrenal glands is a particularly rare event with a poor prognosis.^1^, ^2^ DLBCL should be considered in the differential diagnosis of pineal and adrenal gland tumors. H^1^‐MR spectroscopy provides a noninvasive assessment of lesion metabolism, which makes it a useful adjunct tool.

## ETHICAL STANDARDS

All procedures performed were in accordance with the ethical standards of the institutional research committee and with the 1983 revised Helsinki Declaration and its later amendments or comparable ethical standards.

## CONFLICT OF INTEREST

All authors of this manuscript, Cem Bilgin, Esra Soylu, Baris Korkmaz, Hulya Ozturk, and Kerem Ozturk, declare that they have no conflict of interest.

## AUTHOR CONTRIBUTION

CB, BK, ES, HO, and KO: contributed to the design and implementation of the research, to the analysis of the results, and to the writing of the manuscript. HO: contributed to histopathological sample preparation. Both ES and KO authors contributed to the final version of the manuscript. KO: supervised the article.
